# C-reactive protein in adult sepsis: systematic review and meta-analysis

**DOI:** 10.1016/j.clinsp.2025.100848

**Published:** 2025-12-21

**Authors:** Antonio Silvinato, Clara Lucato dos Santos, Eliane Amorim, Idevaldo Floriano, Luís Eduardo Miranda Paciência, Luca Schiliró Tristão, Wanderley Marques Bernardo

**Affiliations:** a Medicina Baseada em Evidências, Associação Médica Brasileira, São Paulo, SP, Brazil; b Núcleo de Medicina Baseada em Evidências, Unimed Regional da Baixa Mogiana, São Paulo, SP, Brazil; c Departamento de Pediatria, Irmandade da Santa Casa de São Paulo, São Paulo, SP, Brazil; d Federação das Unimeds do Estado de São Paulo (Unimed Fesp), São Paulo, SP, Brazil; e Center for Evidence-Based Medicine, Unimedbxm, São Paulo, SP, Brazil; f Hospital Unimed de Limeira, São Paulo, SP, Brazil; g Obstetric and Gynecology Department, Centro Universitário Faculdade de Medicina do ABC, Santo André, Brazil; h Faculdade de Medicina da Universidade de São Paulo, São Paulo, SP, Brazil; i Faculdade de Medicina da Universidade Lusíada, Santos, SP, Brazil; j Evidence Based Medicine Program of FESP – Coordinator, São Paulo, SP, Brazil

**Keywords:** C-reactive protein, Sepsis, Biomarkers, Diagnosis, Prognosis, Meta-Analysis

## Abstract

*Objective*: To assess the diagnostic and prognostic accuracy of C-Reactive Protein (CRP) in adult patients with suspected or confirmed sepsis.

*Methods*: A systematic review and meta-analysis were conducted using the Medline, Embase, and Google Scholar databases, without language or time restrictions. Studies involving adults at risk for, suspected of, or diagnosed with sepsis who underwent CRP measurement and reported diagnostic or prognostic accuracy data were eligible. Both cross-sectional and cohort studies were included. Risk of bias was evaluated with the QUADAS-2 tool, and the quality of evidence was graded using the GRADE approach.

*Results*: A total of 3599 records were screened, and 22 studies (13,083 patients) met the inclusion criteria, 16 assessing diagnostic accuracy and 6 evaluating prognostic value. In the diagnostic meta-analysis, the pooled sensitivity was 83 % (95 % CI 75 %–89 %) and specificity was 56 % (95 % CI 41 %–69 %), with high heterogeneity (I^2^ = 80.1 %) and a false-positive rate of 44.3 % (95 % CI 30.9 %‒58.6 %). In the prognostic analysis for mortality, the pooled sensitivity was 81 % (95 % CI 70 %–89 %) and the specificity was 77 % (95 % CI 64 %–86 %), with high heterogeneity (I^2^ = 65.9 %) and a false-positive rate of 22.8 % (95 % CI 13.6 %–35.6 %). Most studies presented a moderate to high risk of bias, and the overall quality of the evidence was rated as low.

*Conclusions*: Current evidence reveals high diagnostic uncertainty and limited prognostic accuracy of CRP in adults with sepsis. The low specificity and high error rates restrict the clinical utility of CRP for diagnostic or prognostic decision-making in sepsis management.

## Highlights

• CRP shows high sensitivity but low specificity for adult sepsis diagnosis.
• Diagnostic accuracy of CRP in sepsis is limited by high heterogeneity.
• Prognostic value of CRP for mortality in sepsis remains uncertain.
• False-positive rate of CRP in sepsis diagnosis reaches about 44 %.
• Overall quality of evidence on CRP use in sepsis is low.

## Introduction

C-Reactive Protein (CRP) is an acute-phase protein synthesized by the liver in response to the secretion of several inflammatory cytokines, including Interleukin-6 (IL-6), IL-1 and Tumor Necrosis Factor (TNF). Circulating CRP is produced exclusively by hepatocytes, mainly under transcriptional control by IL-6 and, to a lesser extent, by IL-1β and TNF-α, although other potential sites of local CRP synthesis and secretion have been proposed. *De novo* hepatic synthesis begins rapidly after a single inflammatory stimulus, with serum concentrations exceeding 5 mg/L within approximately six hours and peaking at around 48-hours. The plasma half-life of CRP is approximately 19-hours and remains constant under all physiological and pathological conditions: the circulating CRP level depends solely on the synthesis rate, which reflects the intensity of the pathological processes driving its production. Once the stimulus ceases, CRP levels decline quickly, almost matching the plasma clearance rate. In the general population, CRP concentrations tend to remain stable within individuals, except for transient elevations caused by minor or subclinical infections, inflammation, or trauma.^1^

The biological variability of CRP is critical both for defining appropriate Analytical Performance Specifications (APS) and for accurately interpreting concentration changes in serial measurements. However, this variability remains a matter of concern. Under basal conditions, serum CRP levels are generally stable and extremely low (< 0.5 mg/L) in many individuals. Nevertheless, even minor inflammatory episodes can induce a 10- to 20-fold increase in approximately 25 % of subjects, making it difficult, if not impossible, to assume a true steady state for this protein.^1^

CRP exists in two conformational isoforms: circulating pentameric CRP (pCRP) and monomeric CRP (mCRP), which exert distinct pro- or anti-inflammatory effects. pCRP activates the classical complement pathway, promotes phagocytic activity, and facilitates apoptosis. In contrast, mCRP enhances chemotaxis, recruits leukocytes from circulation to inflammatory sites, and thereby inhibits apoptosis.^1^

Thus, in its native state, CRP exists as a stable pentameric molecule known as pCRP. Upon interacting with activated cell membranes, pCRP undergoes a conformational transition to an activated form (pCRP*), which subsequently dissociates into its monomeric subunits (mCRP). Both pCRP* and mCRP bind to C1q and activate the classical complement pathway, exerting pro-inflammatory effects on platelets and endothelial cells.^1^

CRP is a preferred serological marker for acute inflammatory conditions because of its rapid kinetics and shorter half-life, which lead to a swift decline once inflammation resolves. Its clinical utility has been recognized not only for diagnostic purposes but also for monitoring treatment response.^2^

Sepsis is defined as life-threatening organ dysfunction caused by a dysregulated host response to infection. Sepsis and septic shock remain major global healthcare challenges, affecting millions of people each year and resulting in mortality rates ranging from one in three to one in six. Early recognition and prompt management during the initial hours of sepsis onset significantly improve patient outcomes.^3^

The recommendations presented in this document aim to guide clinicians managing adult patients with sepsis or septic shock in the hospital setting. These guidelines are not intended to replace clinical judgment when caring for individual patients with unique clinical characteristics. Rather, they are designed to reflect current best practices.^3^

For adults with sepsis or septic shock, the authors suggest guiding resuscitation using serum lactate levels to monitor and reduce elevated lactate concentrations, rather than not using lactate assessment. During acute resuscitation, serum lactate values should always be interpreted in the context of the patient’s overall clinical condition and potential alternative causes of elevation. Weak recommendation, low-quality evidence.^3^

For adults with suspected sepsis or septic shock, the authors suggest against using procalcitonin in combination with clinical evaluation to determine when to initiate antimicrobial therapy, as compared with clinical evaluation alone, a poor, very low quality of evidence.^3^

For adults with an established diagnosis of sepsis or septic shock and adequate source control, when the optimal duration of antimicrobial therapy is uncertain, the authors suggest using both procalcitonin AND clinical evaluation to decide when to discontinue antimicrobials, rather than relying solely on clinical evaluation. Weak recommendation, low quality of evidence.^3^

The role of C-reactive protein in adult sepsis can be assessed through a systematic review, which enables estimation of the risk of progression to sepsis among infected patients, determination of its diagnostic accuracy in patients with suspected sepsis, guidance for therapeutic management, and evaluation of the prognosis of patients with established sepsis.

## Methods

### Research question

In adult patients hospitalized with sepsis or suspected sepsis, is the measurement of C-Reactive Protein (CRP) useful as a prognostic, diagnostic, or risk biomarker, or for monitoring therapeutic response?

### Eligibility criteria

A systematic literature review, following the PRISMA Guidelines, was conducted according to the following inclusion criteria:

• Adult patients at risk for, suspected of, or diagnosed with sepsis.
• CRP measurement performed.
• Availability of data on prevalence, risk, or odds ratio, and diagnostic/prognostic accuracy.
• Cross-sectional or cohort study design.
• No restrictions on language or publication period.
• Full-text articles or abstracts containing data of interest.

### Exclusion criterion

• Studies reporting only mean differences.

#### Information sources and search strategy

The databases searched included Medline, Embase, and Google Scholar, complemented by manual.

The search strategies were as follows:

• **Medline:** (Septicemia OR Septicemias OR Sepsis OR "Shock, Septic" OR "Systemic Inflammatory Response Syndrome") AND ("C-Reactive Protein" OR "C Reactive Protein" OR "hs-CRP" OR hsCRP OR "High Sensitivity C-Reactive Protein" OR "High Sensitivity C Reactive Protein") AND ((specificity[Title/Abstract]) OR (prognos*[Title/Abstract] OR (first[Title/Abstract] AND episode[Title/Abstract]) OR cohort[Title/Abstract]));
• **Embase:** ('septicemia'/exp OR septicemia OR septicemias OR 'sepsis'/exp OR sepsis OR 'shock, septic'/exp OR 'shock, septic' OR 'systemic inflammatory response syndrome'/exp OR 'systemic inflammatory response syndrome') AND ('c-reactive protein' OR 'c reactive protein' OR 'hs-crp' OR hscrp OR 'high sensitivity c-reactive protein' OR 'high sensitivity c reactive protein') AND 'diagnostic test accuracy study'/de;
• **Scholar:** Sepsis Sensitivity OR Specificity “C Reactive Protein”.

### Data extraction

The extracted data from each included study comprised study design, patient population, sepsis prevalence, and measures of diagnostic or prognostic accuracy (sensitivity and specificity).

### Quality assessment

The methodological quality and risk of bias of the included studies were evaluated using the QUADAS2^4^ tool, which assesses four domains: patient selection, index test, reference standard, and diagnostic workflow. The risk of bias and quality was classified as low, some concerns, or high using the Robvis visualization tool.^5^ The quality of evidence for each outcome was assessed using the GRADE pro approach and categorized as very low, low, moderate, or high quality of evidence.^6^

### Statistical analysis

The pooled results were summarized through meta-analysis of CRP sensitivity and specificity, and consequently, its error in the diagnostic and prognostic estimation of patients with suspected sepsis or being treated for sepsis. Furthermore, when data on risk prediction or treatment were available, they were incorporated into the analysis when feasible.

Meta-analyses were performed using the Meta-DiSc 2.0 software^7^ in aggregated analyses of sensitivity and specificity expressed in “Forest Plots”; false positive ratios, positive and negative likelihood described numerically; and SROC curve, in univariate and random effect models, with a 95 % confidence level.

## Results

**Fig. 1:**
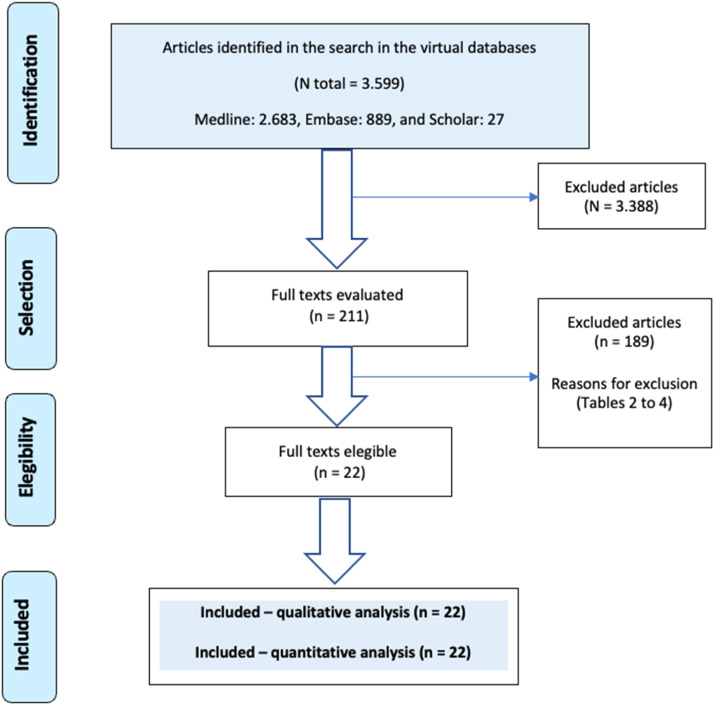
Evidence retrival and selection diagram: PCR & SEPSE.

The database search retrieved a total of 3599 scientific articles (Medline: 2683, Embase: 889, and Scholar: 27). After screening titles and abstracts, 183, 20, and 8 studies were considered potentially eligible. Following full-text assessment, 14-, 6-, and 2-studies were included from Medline, Embase, and Scholar, respectively^8^, ^9^, ^10^, ^11^, ^12^, ^13^, ^14^, ^15^, ^16^, ^17^, ^18^, ^19^, ^20^, ^21^, ^22^, ^23^, ^24^, ^25^, ^26^, ^27^, ^28^, ^29^ (Fig. 1 and Table 1).

**Table 1:** PCR & Sepse – includes.

PMD	First author	Year	Design	Population	Test	Gold standard	Reasons	Sensitivity	Specificity
39,469,063	Daud M	2024	Cohort	Sepsis death (*n* = 59/80)	CRP (98.5 moL)	CRP level	Sepsis prognosis	5 %	68 %
29,731,821	Li Q	2018		Sepsis death (*n* = 26/60)	CRP (no cut off)	Clinical & laboratory criteria	Sepsis prognosis	64.6 %	77.4 %
15,909,277	Póvoa P	2005	Cohort	Sepsis death (*n* = 18/44)	CRP ratio > 0.58	CRP ratio level	Sepsis prognosis	89 %	69 %
7593,905	Yentis SM	1995	Cross sectional	Sepsis death (*n* = 3/18)	CRP (25 % decrease)	CRP level	Sepsis prognosis	97 %	95 %
EMBASE	Karande CB	2023	Cohort	Sepsis death (*n* = 13/50)	CRP (137 moL)	CRP level	Sepsis prognosis	60 %	60 %
EMBASE	Su S	2020	Cohort	Septic shock death (*n* = 21/72)	CRP (no specified)	Clinical & laboratory criteria	Sepsis prognosis	82.2 %	80.3 %
40,214,293	Chuang CL	2025	Systematic review	Sepsis (*n* = 5365/10,755)	CRP 5‒280 mg/L	Clinical & laboratory criteria	Sepsis diagnosis	75 %	68 %
39,844,927	Zhu Q	2024	Cohort	Sepsis (*n* = 289/635)	CRP (> 69 mg/L)	Clinical & laboratory criteria	Sepsis diagnosis	51.1 %	83.6 %
38,025,554	Juneja D	2023	Cohort	Sepsis (*n* = 70/100)	CRP (> 5 mg/L)	Clinical & laboratory criteria	Sepsis diagnosis	98.6 %	3.3 %
35,272,752	Arbutina DD	2022	Cross sectional	SIRS (*n* = 45); Sepsis (*n* = 55)	CRP (no cut off)	Clinical & laboratory criteria	Sepsis diagnosis	80 %	60 %
33,235,597	Sui YD	2020	Cross sectional	Sepsis (*n* = 17/36)	CRP (90.2 mg/L)	Clinical & laboratory criteria	Sepsis diagnosis	52.1 %	61.5 %
27,555,697	Pradhan S	2016	Cross sectional	Sepsis (*n* = 51/64)	CRP (61 mg/L)	Clinical & laboratory criteria	Sepsis diagnosis	84.3 %	53.8 %
25,337,480	Nargis W	2014	Cross sectonal	Sepsis (*n* = 28/73)	CRP (31.4 mg/L)	Clinical & laboratory criteria	Sepsis diagnosis	85.4 %	33.3 %
24,455,636	Meidani M	2013	Cross sectional	Sepsis (*n* = 27/64)	CRP (no specified)	Clinical & laboratory criteria	Sepsis diagnosis	70.5 %	42.1 %
22,056,545	Tsalik EL	2012	Cross sectional	Sepis (*n* = 247/336)	CRP (40 mg/L)	Clinical & laboratory criteria	Sepsis diagnosis	82.3 %	38.7 %
16,407,808	Castelli GP	2006	Cross sectional	Sepis (*n* = 111/255)	CRP (128 mg/L)	Clinical & laboratory criteria	Sepsis diagnosis	61 %	87 %
EMBASE	Wang L	2021	Cross sectional	Sepsis (*n* = 66/90)	CRP (no specified)	Clinical & laboratory criteria	Sepsis diagnosis	87.8 %	58.3 %
EMBASE	Juros GF	2019	Cross sectional	Sepsis (*n* = 36/72)	CRP (18.1 mg/L)	Clinical & laboratory criteria	Sepsis diagnosis	97.2 %	13.9 %
EMBASE	Menon MS	2015	Cross sectional	Sepsis (*n* = 20/94)	CRP (6 mg/L)	Clinical & laboratory criteria	Sepsis diagnosis	93.3 %	63.3 %
EMBASE	Farag NA	2013	Cross sectional	Sepsis (*n* = 15/80)	CRP (51 mg/L)	Clinical & laboratory criteria	Sepsis diagnosis	93 %	80 %
SCHOLAR	Zhang H	2017	Cross sectional	Sepsis (*n* = 50/70)	hs-CRP (74.2 mg/L)	Clinical & laboratory criteria	Sepsis diagnosis	78 %	75 %
SCHOLAR	Chen M	2017	Cross sectional	Sepsis (*n* = 77/127)	hs-CRP (75 mg/L)	Clinical & laboratory criteria	Sepsis diagnosis	81.2 %	68.2 %

hs-CRP, High-Senstivity C-Reactive Protein.

The final sample comprised 22 studies, distributed as follows: 16 diagnostic and 6 prognostic investigations. Study designs included 7 cohort studies, 14 cross-sectional studies, and 1 systematic review with meta-analysis of 44 studies, resulting in a total of 65 primary studies analyzed. Among the excluded studies, the most common reasons were studies evaluating infections other than sepsis and those focused only on absolute CRP level without assessing diagnosis accuracy. Also, several articles analyze derived ratios such as CRP/albumin, CRP/platelet, and so on. Studies involving other populations rather than adults with sepsis as pediatric populations, or others with insufficient data, or outcomes different than those established in this systematic review criteria.

The total number of participants was 13,083; of whom 12,901 were included in diagnostic analyses and 182 in prognostic analyses. Diagnostic studies compared septic from non-septic patients, whereas prognostic studies evaluated mortality outcomes. No studies evaluating sepsis risk or therapeutic monitoring were included.

The index test in all studies was C-Reactive Protein (CRP), with defined cutoff values ranging from 5 mg/L to 280 mg/L. The reference standards varied according to clinical and/or laboratory criteria (Table 1).

## Diagnosis

**Fig. 2:**
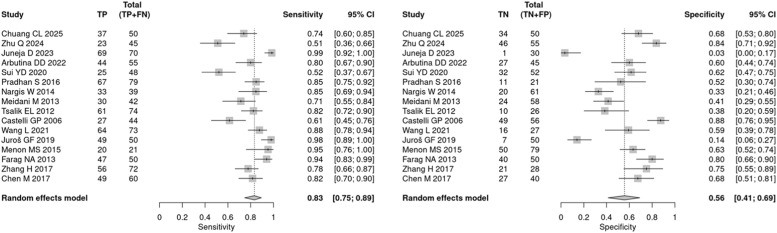
CPR diagnostic sensitivity and specificity.

In the diagnostic meta-analysis, the mean prevalence (pre-test probability) of sepsis was 54.4 %. The pooled sensitivity was 83 % (95 % CI 75 % to 89 %) and specificity was 56 % (95 % CI 41 % to 69 %) (Fig. 2). Heterogeneity was substantial (I^2^ = 80.1 %) in the bivariate analysis.

The false-positive rates were 44.3 %, indicating that across varying cutoff thresholds, the estimated probability of erroneously classifying a patient as septic based on a positive CPR result was 44.3 % (95 % CI 30.9 % to 58.6 %).

**Fig. 3:**
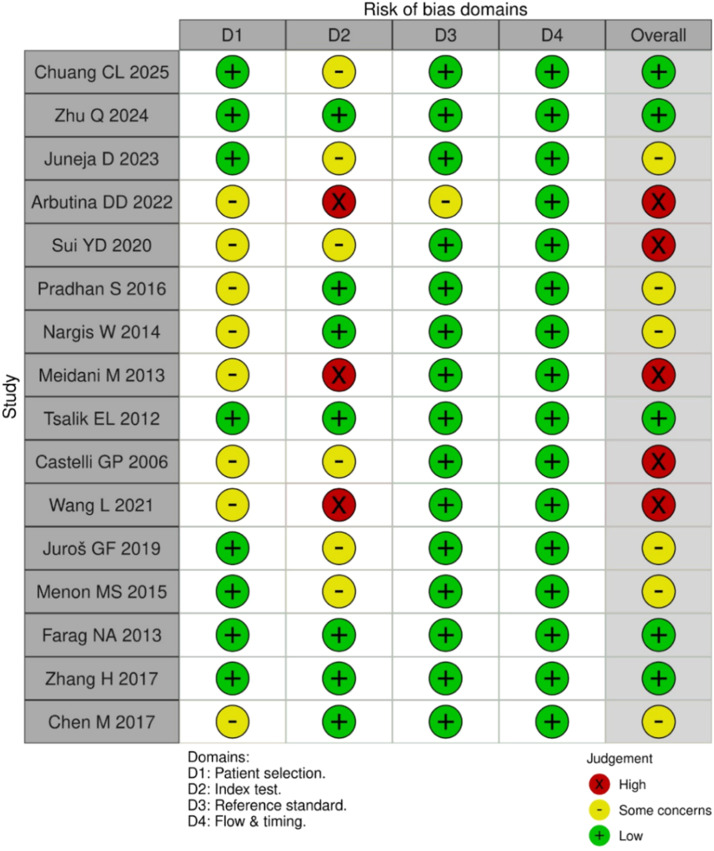
Diagnosis studies bias.

The risk of bias was rated as very high in 31 %, high in 37 %, and low in 32 % of the included studies (Fig. 3). According to the GRADE assessment, the overall quality of evidence was classified as low (Table 2).

**Table 2:** Quality of evidence of diagnosis studies ‒ GRADE.

Outcome	N° of studies (N° of patients)	Study design	Factors that may decrease certainty of evidence					Effect per 1.000 patients tested	Test accuracy CoE
			Risk of bias	Indirectness	Inconsistency	Imprecision	Publication bias	Pre-test probability of 54.4 %	
True positives (patients with SEPSE)	16 studies	Cross-sectional (cohort type accuracy study)	Serious^a^^,^^b^	Not serious	Serious^c^	Not serious	None	452 (408 to 484)	⨁⨁◯◯ Low^a^^,^^b^^,^^c^
False negatives (patients incorrectly classified as not having SEPSE)	7018 patients							92 (60 to 136)	
True negatives (patients without SEPSE)	16 studies	Cross-sectional (cohort type accuracy study)	Serious^a^^,^^b^	Not serious	Serious^c^	Not serious	None	255 (187 to 315)	⨁⨁◯◯ Low^a^^,^^b^^,^^c^
False positives (patients incorrectly classified as having SEPSE)	5883 patients							201 (141 to 269)	

Explanations:.

a Selection bias.

b Index test bias.

c Heterogeneity > 50 %.

## Prognosis

**Fig. 4:**
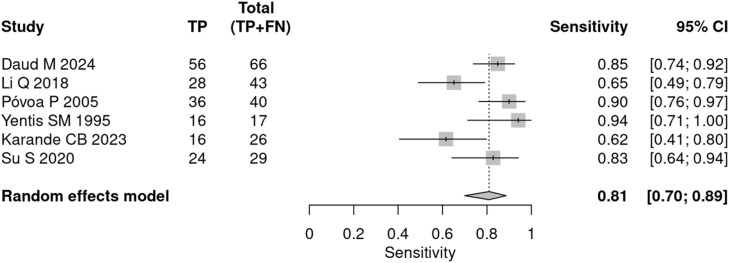
CPR prognostic sensitivity.

**Fig. 5:**
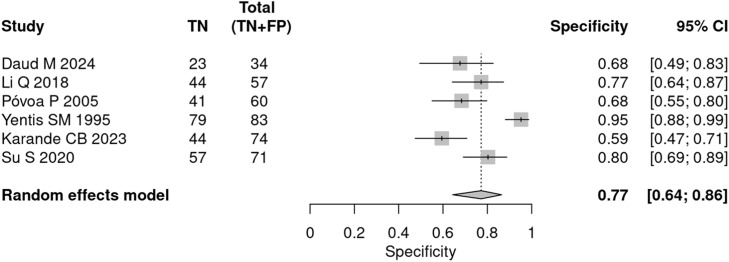
CPR prognostic specificity.

In the prognostic meta-analysis, the mean prevalence (pre-test probability) of mortality in patients with sepsis was 36.9 %. The pooled sensitivity was 81 % (95 % CI 70 % to 89 %), and the specificity was 77 % (95 % CI 64 % to 86 %) (Fig. 4, Fig. 5). Heterogeneity was moderate to high (I^2^ = 65.9 %) in the bivariate analysis.

The false-positive rate was 22.8 %, indicating that, across different cutoff values, the estimated error in considering a positive CPR in the prognostic estimate of mortality in sepsis was 22.8 % (95 % CI 13.6 % to 35.6 %).

**Fig. 6:**
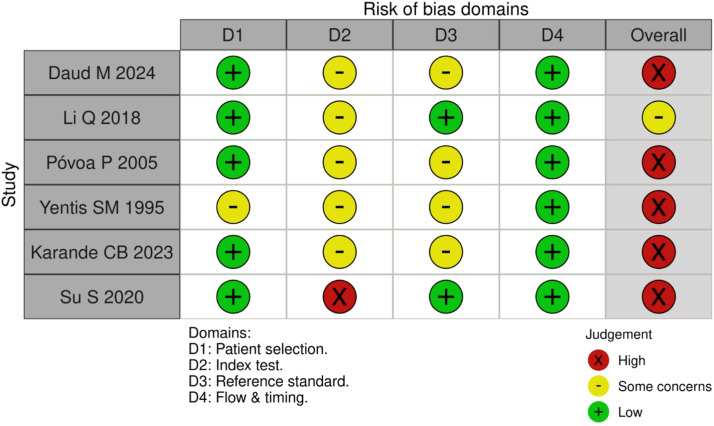
Prognosis studies bias.

The risk of bias was classified as very high and high in 83 % and 17 % of the included studies, respectively (Fig. 6). The overall quality of the evidence was rated as low (Table 3).

**Table 3:** Quality of evidence of prognosis studies – GRADE.

Outcome	N° of studies (N° of patients)	Study design	Factors that may decrease certainty of evidence					Effect per 1.000 patients tested	Test accuracy CoE
			Risk of bias	Indirectness	Inconsistency	Imprecision	Publication bias	Pre-test probability of 36.9 %	
True positives (patients with DEATH)	6 studies	Cross-sectional (cohort type accuracy study)	Serious	Not serious	Serious	Not serious	None	299 (258 to 328)	⨁⨁◯◯ Low
False negatives (patients incorrectly classified as not having DEATH)	67 patients							70 (41 to 111)	
True negatives (patients without DEATH)	6 studies	Cross-sectional (cohort type accuracy study)	Serious	Not serious	Serious	Not serious	None	486 (404 to 543)	⨁⨁◯◯ Low
False positives (patients incorrectly classified as having DEATH)	115 patients							145 (88 to 227)	

## Discussion

There are two main reasons behind the widespread use of this test. The first is the broad spectrum of clinical conditions in which CRP provides useful information. The second is its analytical robustness: CRP yields consistent results in fresh, stored, or frozen samples, it is unaffected by food intake, presents negligible diurnal and seasonal variation, and has a well-definite half-life. Furthermore, CRP concentrations can be measured using automated, low-cost assays that are widely available in clinical laboratories across high-, middle-, and low-income countries.^1^

Despite these advantages, the volume and variability of CRP test requests remain a matter of concern. Efforts to improve appropriateness and to avoid unnecessary and repeated testing have included the introduction of Minimum Retesting Interval (MRI), a demand-management strategy to identify and reduce overused laboratory tests. Guidelines recommend that CRP should not be repeated within a 24-hour period, with the exception of requests in neonates, and that automated, IT-based systems enforcing a 48-hours retesting rule can significantly reduce redundant testing. Such measures yield cost savings, improve laboratory efficiency, and maintain the quality and safety of patient care.^1^

Historically, CRP measurement in serum or plasma evolved from qualitative to semiquantitative, and eventually to fully quantitative assays. Early techniques included Radial Immunodiffusion (RID), agarose gel electrophoresis, and Latex-Agglutination (LA). Later on, Electro Immunoassay (EIA), Immunoturbidimetric (IT), Laser Nephelometry (LN), and Immunofluorimetric (IF) methods. Advances in analytical technology have increased sensitivity and markedly reduced Turnaround Time (TAT). More recently, Point-of-Care Testing (POCT) has been implemented to guide prescribing, particularly for lower respiratory tract infections.^1^

CRP elevation has been documented in several pathological conditions, including infections, malignancies, ischemic necrosis, and trauma. However, there is evidence that CRP levels rise modestly despite active tissue-damaging inflammatory processes in some disorders, including systemic lupus erythematosus, scleroderma, dermatomyositis, Sjögren’s syndrome, ulcerative colitis, graft-versus-host disease and leukemia.^1^

A growing body of evidence links inflammation to Cardiovascular Diseases (CVD), including coronary heart disease, ischemic stroke, and acute myocardial infarction, as well as Peripheral Vascular Diseases (PVD). The prospect of using CRP as a predictor of future vascular risks was limited because existing assay methods, such as latex agglutination and capillary immunoprecipitation with 3–8 mg/L of a Limit of Detection (LOD), were not sensitive enough to detect very low levels of CRP in serum. High-sensitivity CRP (hs-CRP) assays were subsequently developed, providing detection limits around 0.00016 mg/L and analytical Coefficients of Variation (CV) below 15 % at 0.2 mg/L. These advances enabled accurate quantification of very low serum CRP levels and strengthened the evidence linking CRP with the incidence of major Coronary Heart Disease (CHD) events.^1^

Inflammation is increasingly recognized as a central mechanism underlying numerous diseases, including chronic conditions such as neurodegenerative disorders. CRP is a major focus of research and is fast becoming one of the most extensively studied plasma proteins in humans. Emerging evidence indicates that CRP is not merely an inflammatory marker but also a potential mediator of inflammation, playing a complex role in chronic inflammatory states and their associated pathologies. This is particularly relevant to neurodegenerative diseases, which involve progressive neuronal dysfunction and persistent inflammation. Neurodegenerative proteinopathies are characterized by the accumulation of misfolded protein aggregates that induce cellular toxicity and proteostatic collapse. Misfolded proteins can be deposited in tissues in the form of amyloid fibrils and cause progressive organ dysfunction.^2^

The present findings demonstrate a prohibitively high diagnostic error rate (approximately 44 % false positives) when CRP is used as a biomarker for sepsis diagnosis in adults. Notably, the Surviving Sepsis Campaign guidelines^3^ do not recommend CRP for diagnostic management, reserving this role for serum lactate and, obviously, clinical practice.

Although the error rate was lower (22 %) when CRP was evaluated as a prognostic biomarker (mortality), the limited number of studies conducted to date (only 180 patients studied) is striking, as is the low quality of evidence, such as its use as a diagnostic biomarker.

The difference in characteristics among the studies led to high heterogeneity, reducing the strength of the evidence. Future studies should use a cutoff that is, if not identical, at least more similar. Furthermore, the large variation in prevalence across studies ‒ something the author cannot control ‒ affects the sensitivity of the test. Most studies had a high or very high risk of bias. All these factors led to a low certainty of evidence, thus justifying the present study’s conclusion.

The literature frequently employs continuous variables, such as mean differences between different forms of investigation, such as risk, accuracy, prognosis, or therapeutic management. However, such approaches are not ideal for CRP, given its nonspecific responsiveness to inflammation, its elevation in many non-infectious conditions, and the ease with which small but statistically significant differences can emerge, potentially amplifying diagnostic error. Furthermore, continuous analyses do not allow direct estimation of error magnitude when using CRP as a biomarker.

It is essential to recognize that the conclusions of a systematic review, based on the analysis of adult sepsis cases and the use of the CRP biomarker, partially and spontaneously published, cannot replace the clinician's decision-making ability when faced with a patient's specific clinical variables. Furthermore, it is always possible and recommended for different healthcare settings to record and analyze their own data, as this is the only way to validate or not the conclusions obtained indirectly through the analysis of published literature on the subject of interest.

## Conclusions

To date, evidence points to the highly uncertain nature of the use of C-Reactive Protein (CRP) in the diagnostic management or prognostic assessment of adult patients with suspected or already diagnosed sepsis. Estimated errors (false positives) range from approximately 30 % to 58 % (mean 44 %), and from 13 % to 35 % (mean 22 %), in diagnostic and prognostic accuracy analyses, respectively.

## Data availability

All data supporting the findings of this study are available within the article.

## CRediT authorship contribution statement

**Antonio Silvinato:** Conceptualization, Investigation, Writing – original draft. **Clara Lucato dos Santos:** Writing – review & editing, Visualization. **Eliane Amorim:** Conceptualization, Writing – review & editing. **Idevaldo Floriano:** Investigation, Formal analysis, Writing – original draft. **Luís Eduardo Miranda Paciência:** Conceptualization, Writing – review & editing. **Luca Schiliró Tristão:** Writing – review & editing, Visualization. **Wanderley Marques Bernardo:** Conceptualization, Methodology, Supervision, Project administration.

## Declaration of competing interest

The authors declare no conflicts of interest.
